# The Role of the E3 Ligase Cbl-B in Murine Dendritic Cells

**DOI:** 10.1371/journal.pone.0065178

**Published:** 2013-06-06

**Authors:** Stephanie Wallner, Christina Lutz-Nicoladoni, Christoph H. Tripp, Günther Gastl, Gottfried Baier, Josef M. Penninger, Patrizia Stoitzner, Dominik Wolf

**Affiliations:** 1 Laboratory of Tumor Immunology, Tyrolean Cancer Research Institute, Innsbruck, Austria; 2 Internal Medicine V, Haematology and Oncology, Innsbruck, Austria; 3 Department of Dermatology and Venereology, Medical University Innsbruck, Innsbruck, Austria; 4 Department for Medical Genetics, Molecular and Clinical Pharmacology, Medical University Innsbruck, Innsbruck, Austria; 5 IMBA, Institute of Molecular Biotechnology of the Austrian Academy of Sciences, Vienna, Austria; 6 Department of Hematology and Oncology, University Hospital Bonn, Bonn, Germany; University Hospital of Heidelberg, Germany

## Abstract

Dendritic cells (DCs) are potent antigen-presenting cells with a promising potential in cancer immunotherapy. Cbl proteins are E3 ubiquitin ligases and have been implicated in regulating the functional activity of various immune cells. As an example, c-Cbl negatively affects DC activation. We here describe that another member of the Cbl-protein family (i.e. Cbl-b) is highly expressed in murine bone-marrow-derived DCs (BMDCs). Differentiation of *cblb*−/− bone marrow mononuclear cells into classical BMDCs is unaltered, except enhanced induction of DEC-205 (CD205) expression. When tested in mixed-lymphocyte reaction (MLR), *cblb*−/− BMDCs exhibit increased allo-stimulatory capacity *in vitro*. BMDCs were next *in vitro* stimulated by various toll like receptor (TLR)-agonists (LPS, Poly(I:C), CpG) and exposed to FITC-labeled dextran. Upon TLR-stimulation, *cblb*−/− BMDCs produce higher levels of proinflammatory cytokines (IL-1α, IL-6 and TNF-α) and exhibit a slightly higher level of FITC-dextran uptake. To further characterize the functional significance of *cblb*−/− BMDCs we tested them in antigen-specific T cell responses against ovalbumin (OVA) protein and peptides, activating either CD8^+^ OT-I or CD4^+^ OT-II transgenic T cells. However, *cblb*−/− BMDCs are equally effective in inducing antigen-specific T cell responses when compared to wildtype BMDCs both *in vitro* and *in vivo*. The migratory capacity into lymph nodes during inflammation was similarly not affected by the absence of Cbl-b. In line with these observations, *cblb*−/− peptide-pulsed BMDCs are equally effective vaccines against OVA-expressing B16 tumors *in vivo* when compared to wildtype BMDCs. We conclude that in contrast to c-Cbl, Cbl-b plays only a limited role in the induction of Ag-specific T cell responses by murine BMDCs *in vitro* and *in vivo*.

## Introduction

DCs are a unique class of leukocytes whose primary function is to capture antigens and process them to activate or tolerize T cells [1]. Thus, the high efficacy of DCs in initiating primary T cell responses inspired tumor immunologists to evaluate the therapeutic potential of DCs as cellular vaccine to induce antigen-specific immune responses targeting cancer cell associated antigens [2]. However, despite huge efforts to establish DC vaccination as a treatment for cancer patients, therapeutic success is limited so far. Nevertheless, a randomized trial in hormone-refractory prostate cancer led to the first FDA approval of a DC-based vaccine after observation of an improved overall survival rate of DC-treated patients, whereas numerous other studies (i.e. in melanoma etc.) did not yield positive clinical results [3], [4]. Thus, at least in the current application schedules DC vaccines do not sufficiently help cancer patients by inducing significant clinical responses, in spite of consistently measurable T cell responses and a virtual lack of therapy-related side-effects [5], [6]. Therefore improvement of DC vaccines is a clear unmet medical need.

Casitas b-linage lymphoma proto-oncogene b (Cbl-b) is a member of a highly evolutionary conserved family of Cbl proteins, which in mammals consists of three Cbl genes: Cbl (also termed c-Cbl, Cbl2, RNF55), Cbl-b (also termed RNF56) and Cbl-c/Cbl-3 (also termed, Cbl-SL, RNF57) [7], [8]. Cbl proteins interact with target proteins *via* their protein–protein interaction domains, allowing regulation of multiple signaling pathways in immune cells [8].

The E3 ubiquitin ligase c-Cbl, known for its roles in regulating lymphocyte signaling has now been identified to be a modulator of DC activation as well [9]. In *ccbl*−/− DCs, Toll-like receptor (TLR)-induced expression of proinflammatory cytokines such as IL-12p70, IL-6, IL-1α and IL-1β was significantly enhanced. This correlated with a greater potency of DC-based vaccines against established tumors when *ccbl*−/− DCs were used [9]. A recent study indicates that the E3 ubiquitin ligase Cbl-b plays a critical role in the regulation of TLR-triggered inflammatory response *via* degradation of MyD88, which is a central component of TLR-signaling [10]–[13]. Additionally, the interaction between programmed cell death ligand 1 (PD-L1) on DCs and programmed cell death 1 (PD-1) on CD8^+^ T cells contributes to ligand-induced TCR down-modulation, which occurs *via* Cbl-b up-regulation in CD8^+^ T cells. PD-L1/PD-1 interference enhances CD8^+^ T cell anti-tumor activities by interfering with Cbl-b up-regulation [14], [15]. Further studies demonstrated that blocking of PD-1 causes reduced Cbl-b expression and blocking of PD-L1/PD-1 blockade is also showing very promising results in the clinic [16], [17]. So far, the role of Cbl-b in DC biology has not been addressed. Substantial evidence already supports an important role for Cbl-b in various cells of the immune system. More precisely, Cbl-b has been described as a critical gatekeeper of anergy avoidance pathway in T and NKT cells [18]–[23]. This knowledge has recently been used to improve the efficacy of adoptive T cell therapy by using *cblb*−/− T cells as cellular therapy, as this renders T cells resistant to inhibitory cues of the tumor microenvironment allowing improved cancer regression rates [24]–[27]. According to this idea, we recently published that the adoptive transfer of *cblb*−/− CD8^+^ T cells in combination with a DC vaccination in immune-competent mice significantly delays tumor growth in various mouse tumor models [28]. Moreover, at least in defined cellular systems such as osteoclastogenesis or thymocyte development Cbl-b and c-Cbl have redundant functions [29], [30]. These data support the idea, that targeting the Cbl-b pathway may be a promising strategy to enhance anti-tumor efficacy by potentially not only interfering with T cell but also by modulating DC biology. Based on these ideas we systematically tested impact of *cblb*-deficiency on the biological behaviour of murine DCs to finally evaluate whether anti-tumor responses could further be improved by using *cblb*−/− DCs instead of wildtype DCs for vaccination.

## Materials and Methods

### Mice

*Cblb* mutant mice were crossed into a C57BL/6 background and genotyped by PCR. Littermate control groups were used and mice of inbred strains C57BL/6, BALB/c, C57BL/6-OT-I and C57BL/6-OT-II were purchased from Charles River Laboratories (Germany) and used at the age of 7–12 weeks, sex and age matched. T cells from OT-I and OT-II mice express a transgenic Vα2Vβ5.1/5.2 T cell receptor (TCR) specific for the OVA peptides presented on H2-Kb (amino acids 257 to 264; SIINFEKL) or on I-Ab (amino acids 323 to 339; ISQAVHAAHAEINEAGR), respectively. Mice were maintained under specific pathogen-free conditions. This study was carried out in strict accordance with protocols approved by the Institutional Animal Care and Use Committee of the Medical University Innsbruck (Permit Numbers: 66011/116-II/10b/2008, 66011/146-II/3b/2012). All surgery was performed under anesthesia, and all efforts were made to minimize suffering.

### Generation of BMDCs

BMDCs were generated following a modified protocol described earlier [31]. Briefly, bone marrow was collected by flushing the tibia and femora of *cblb*−/− and wildtype mice, cultured in RPMI supplemented with 10% fetal calf serum, 2 mM L-glutamine, 100 U/ml penicillin, 0.1 mg/ml streptomycin, 0.05 mM β-mercaptoethanol and 200 U/ml granulocyte-monocyte colony-stimulating factor (GM-CSF), obtained from supernatant of X38-Ag8 plasmacytoma cells (kind gift of A. Lanzavecchia, Bellinzona, CH). For *in vitro* studies immature (not exposed to LPS) and mature DCs induced by incubating day seven non-adherent BMDCs with 100 ng/ml LPS (Sigma Aldrich) over night were used. For *in vivo* vaccination studies we used semi-mature BMDCs exposed for 4 hours to LPS.

### Flow Cytometric Analysis of Cell Surface Marker

Cell suspensions from spleen and lymph nodes were prepared by teasing the organs into small fragments with scissors followed by digestion with 0.25 mg/mL collagenase P (Roche Diagnostics) and 120 µg/mL DNase I (Roche Diagnostics) for 30 minutes at 37°C. After digestion tissue was pressed through cell strainers (70 µm, BD Falcon), essentially as described recently for spleen cell suspensions [32]. Flow cytometry analyses were performed to analyze BMDCs from wildtype and *cblb*−/− mice. Briefly, 1×10^6^ cells in 100 µL of flow buffer (ice-cold PBS plus 1% FCS) were stained with fluorescently-labeled cell surface-specific primary antibodies for 30 min at 4°C. Cells were washed twice with flow buffer. All flow cytometry data were acquired using a multi-colour flow cytometer (FACSCalibur or FACSAria, BD Biosciences) equipped with the CellQuest Pro™ or FACSDiva™ software (BD Biosciences). Staining was performed with following, directly labeled, monoclonal antibodies: MHC II/I-A/I-E-PE-Cy7 (clone M5/114.15.2) antibody was purchased from eBioscience, CD11c-PerCP (clone N418), CD205/DEC-205-APC (clone NLDC-145), CD83-FITC (clone Michel-19), MHC II/I-A/I-E-PerCP (clone M5/114.15.2), CD80-PerCP/Cy5.5 (clone 16-10A1), CD40-PE (clone 3/23), CD14-PE/Cy7 (clone Sa14-2), CCR7-APC (clone 4B12) from Biolegend and Macrophage Mannose receptor/CD206-Alexa Fluor 647 (clone MR5D3) from Serotec. Data analysis was performed using FlowJo™ software (Tree Star).

### Isolation of CD4 and CD8 T cells

CD8^+^ and CD4^+^OVA-specific T cells were isolated from spleen and lymph node suspensions obtained from OT-I and OT-II transgenic mice by using magnetic bead separation (CD4^+^ T cell isolation kit; CD8^+^ T cell isolation kit; Miltenyi Biotec). Briefly, non-CD4^+^ or CD8^+^ T cells were depleted using a biotinylated antibody cocktail. The purity of both populations was assessed by flow cytometric analysis and routinely yielded a purity of 85–95%. The T cells from BALB/c mice for MLR were prepared by passing spleen suspensions through cell strainer and T cells were isolated with magnetic beads (Pan T cell isolation kit; Miltenyi Biotec).

### *In vitro* Antigen-specific T cell Proliferation

CD8^+^ and CD4^+^ OVA-specific T cells were isolated from OT-I and OT-II transgenic mice, respectively. Wildtype and *cblb*−/− BMDCs were matured with 100 ng/ml LPS and primed with SIINFEKL peptide (amino acids 257 to 264; SIINFEKL; Proimmune) or I-Ab peptide (amino acids 323 to 339; ISQAVHAAHAEINEAGR; AnaSpec Inc.) overnight. BMDCs were applied in graded doses in triplicates to 2×10^5^ transgenic CD8^+^ or CD4^+^ T cells in 96-well round-bottomed culture plates for 48 hours. Proliferation of T cells was measured by uptake of 1 µCi ^3^H-Thymidine (Hartmann Analytik Braunschweig, Germany) pulsed for the last 16–18 hours of incubation by using a β-scintillator (MicroBeta TriLux, Perkin Elmer).

### *In vivo* Antigen-specific T cell Proliferation Assay

CD8^+^OVA-specific T cells were enriched from suspensions of lymph nodes and spleens of OT-I transgenic mice by negative selection (Miltenyi Biotec). CD8^+^ T cells were labeled at 2×10^6^ cells/ml with 2 µM carboxy-fluorescein diacetate succinimidylester (CFSE; Invitrogen-Molecular Probes) for 10 min at 37°C. The reaction was quenched with an equal volume of FCS and cells were washed three times with PBS/0.1% BSA. C57BL/6 recipient mice were injected intravenously with 5×10^6^ CFSE-labeled transgenic CD8^+^ T cells. 24 hours thereafter we subcutaneously injected 5×10^6^ BMDCs which had been matured with 100 ng/ml LPS and pulsed *in vitro* with or without 10 µM SIINFEKL peptide (Proimmune) at 37°C overnight. Cell suspensions were prepared from draining (inguinal) and non-draining lymph node 72 hours later by pressing the lymph nodes through cell strainers with the plunger of a syringe. Groups of 3 mice per treatment were used routinely. Proliferation of antigen-specific CD8^+^ CFSE^+^ T cells was detected by the decrease of CFSE fluorescence intensity as described earlier [33].

### Cross-presentation-Assay

CD8^+^ and CD4^+^ OVA-specific T cells were isolated from OT-I and OT-II transgenic mice respectively. Wildtype and *cblb*−/− BMDCs were matured with 100 ng/ml LPS and loaded with 1 mg/ml OVA-protein (Sigma Aldrich) overnight. BMDCs were applied in graded doses in triplicates to 2×10^5^ freshly prepared OT-I transgenic CD8^+^ and OT-II transgenic CD4^+^ T cells in 96-well round-bottomed culture plates for 48 hours. Proliferation of T cells was measured by uptake of 1 µCi ^3^H-Thymidine (Hartmann Analytik) pulsed for the last 16–18 hours of incubation.

### Mixed Lymphocyte Reaction

For MLR experiments, suspensions of responder T cells were cultured with mature allogeneic stimulator BMDCs. BMDCs of C57BL/6 mice were matured with 100 ng/ml LPS for 24 hours. T cells were purified from the spleens of BALB/c mice by negative magnetic bead isolation (Pan T cell isolation kit, Miltenyi Biotec). Allogeneic stimulator cells were cultured at different numbers in triplicates with 2×10^5^ T cells in 96-well round-bottomed culture plates for 48 hours. Proliferation of T cells was measured by uptake of 1 µCi ^3^H-Thymidine (Hartmann Analytik) pulsed for the last 16–18 hours of incubation.

### Macropinocytosis

For the measurement of macropinocytosis immature BMDCs were washed and subsequently resuspended in PBS for incubation with 2 mg/ml FITC-dextran (Sigma Aldrich) at 37°C or at 4°C (negative control). After 2 and 4 hours, uptake was terminated by adding ice cold PBS containing 2% FCS and 0.01% NaN_3_. Cells were washed a further four times and analyzed by flow cytometry. Surface binding values obtained when incubating cells at 4°C were subtracted from values measured at 37°C.

### Cytokine Secretion

Immature 1×10^5^ wildtype and *cblb*−/− BMDCs were stimulated in triplicates with 1 µg/ml TLR4 agonist (LPS; Sigma Aldrich), 20 µg/ml TLR3 agonist (Poly(I:C); Imgenex) or 100 nM TLR9 agonist (CpG-ODN; TIB Molbiol) on day seven of *in vitro* culture. After 24 hours, culture supernatants were collected and analyzed for IL-12p70, IL-10, IFN-γ, IL-1α, IL-6, TNF-α, KC, MIP-1α and MCP-1, according to the manufacturer’s manual by Bioplex (Bio-Plex Pro™ Mouse Cytokine Standard; 23-Plex, Group I, Biorad).

### *In vivo* BMDCs Migration

Mycobacterium tuberculosis (TB; fc = 0.125 mg; Voigt Global Distribution Inc.) in Freund’s incomplete adjuvants (FIA; Sigma Aldrich) was subcutaneously injected into the hock of 7–12 weeks old female wildtype recipients under anesthesia [34]. Two days after TB application, 5×10^6^ 2 µM CFSE (Invitrogen-Molecular Probes) labeled immature wildtype BMDCs and 6 µg/ml Tetramethylrhodamine (TAMRA; Invitrogen-Molecular Probes) labeled immature *cblb*−/− BMDCs were subcutaneously injected in the hock of TB-FIA injected recipients and solvent injected wildtype recipients as control for spontaneous migration under anesthesia. The total injection volume in the hock was a maximum of 50 µl. 24 hours thereafter migration of fluorescent-labeled BMDCs in the draining (popliteal) lymph node, inguinal lymph node and spleen was measured by flow cytometry.

### *In vivo* Tumor Model

OVA-expressing B16 tumors [35] (a kind gift of Dr. E.M. Lord and Dr. J.G. Frelinger, University of Rochester, Rochester, NY, USA)were induced by subcutaneous injection of 1×10^5^ tumor cells in the left flank of female wildtype C57BL/6 mice. *Cblb*−/− or wildtype BMDCs were pulsed with 10 µM SIINFEKL peptide (Proimmune) and stimulated with 100 ng/ml LPS for 4 hours. 2×10^5^ SIINFEKL-loaded wildtype or *cblb*−/− BMDCs were subcutaneously injected into the contralateral flank of tumor-bearing mice, five days after tumor challenge. In all experimental groups, tumor growth was monitored three times per week by measuring tumor length and width with a caliper. Tumor volume was calculated as: ((length×width^2^) × π))/6000. For survival analysis, mice with tumors above the length limit of 20 mm were sacrificed.

### Western Blotting

BMDCs were lysed in whole cell lysis buffer as described in [36]. After clearance of the homogenates by centrifugation, 15–30 µg of cell-lysate were resolved on 4–12% SDS page using bis-tris-buffered polyacrylamide gels. Proteins are electro-transferred to polyvinylidene difluoride membranes. Membranes were then blocked in 5% milk and probed with anti-Cbl-b (Abcam) and anti-β-Actin (Santa Cruz), detected with HRP-conjugated secondary antibodies and enhanced chemoluminescence.

### Statistical Analysis

The statistical analysis of the difference between mean values was performed using the paired Student’s t-test. Overall survival is expressed by the Kaplan-Meier method. P-values expressed as **p<0.005 or *p<0.05 were considered statistically significant. Numbers of carried out experiments are indicated in figure legends.

## Results

### Cbl-b is Highly Expressed in BMDCs and does not Affect BMDCs Differentiation *in vitro*

**Figure 1:**
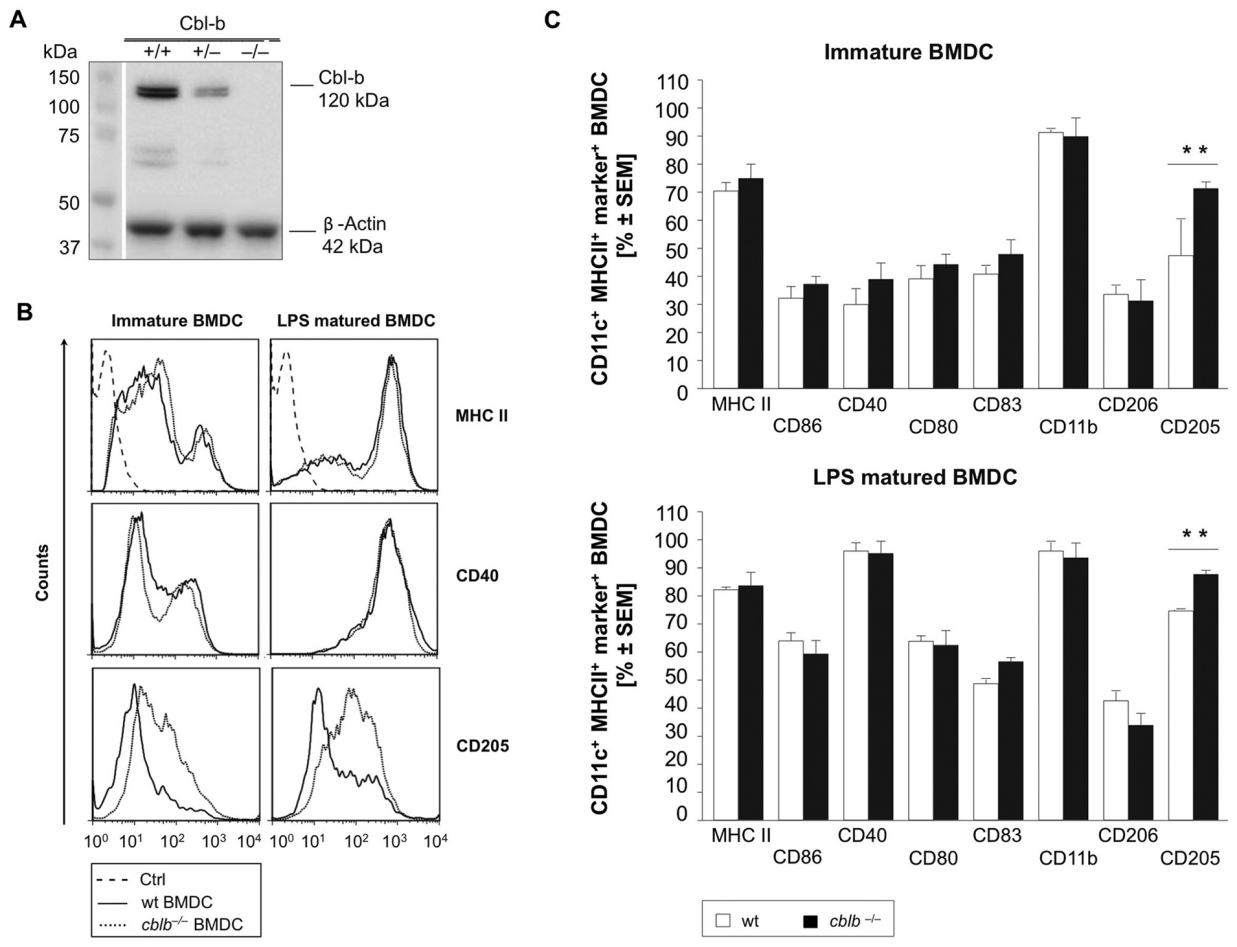
Cbl-b is highly expressed in BMDCs and does not affect DC differentiation. (A) Cbl-b Protein expression of *in vitro* generated 85–95% pure immature BMDCs determined by Western blotting. (B) Surface marker expression of wildtype and *cblb*−/− BMDCs was analyzed by multi-colour flow cytometry. The overlay histograms depict the expression of wildtype *versus cblb*−/− BMDC surface markers of immature cells after seven days of culture and LPS matured cells after eight days of culture. Representative FACS plots, n = 6 mice per group. The graphs in (C) depict the percentage of CD11c^+^ MHC-II^+^ and surface marker positive BMDCs either immature cells at day seven of culture or LPS matured cells at day eight of culture. Data represent mean value ± SEM; n = 6, **p<0.005.

We first evaluated, whether Cbl-b is expressed in BMDCs. Therefore we analyzed protein expression of Cbl-b by Western blot using *in vitro* generated BMDCs. Cbl-b protein is readily detectable in highly pure (80–90% CD11c^+^ MHCII^+^) BMDCs but absent in *cblb*−/− BMDCs (Figure 1A). We next tested whether Cbl-b deficiency affects BMDC differentiation. However, no difference in BMDCs cell yields on day seven of *in vitro* GM-CSF mediated differentiation could be detected between wildtype and *cblb*−/− BMDCs (data not shown). This, however, does not exclude that *cblb*−/− BMDCs display a different phenotype.

### DEC-205 is Specifically Up-regulated in *cblb*−/− BMDCs

We next investigated the surface marker expression of wildtype and *cblb*−/− BMDCs. Immature and LPS-stimulated mature BMDCs were analyzed for the expression of common DC surface markers by flow cytometry. Expression of BMDC markers, such as MHC class II, CD86, CD40, CD80, CD83, CD11b and macrophage mannose receptor/CD206 were not differentially regulated in *cblb*−/− *versus* wildtype BMDCs (Figure 1 B/C). However, we found a consistent increase of DEC-205 expression in *cblb*−/− BMDCs when compared to wildtype BMDCs (Figure 1 B/C).

### Enhanced Proinflammatory Cytokine and Chemokine Secretion of *cblb*−/− BMDCs upon TLR Stimulation

**Figure 2:**
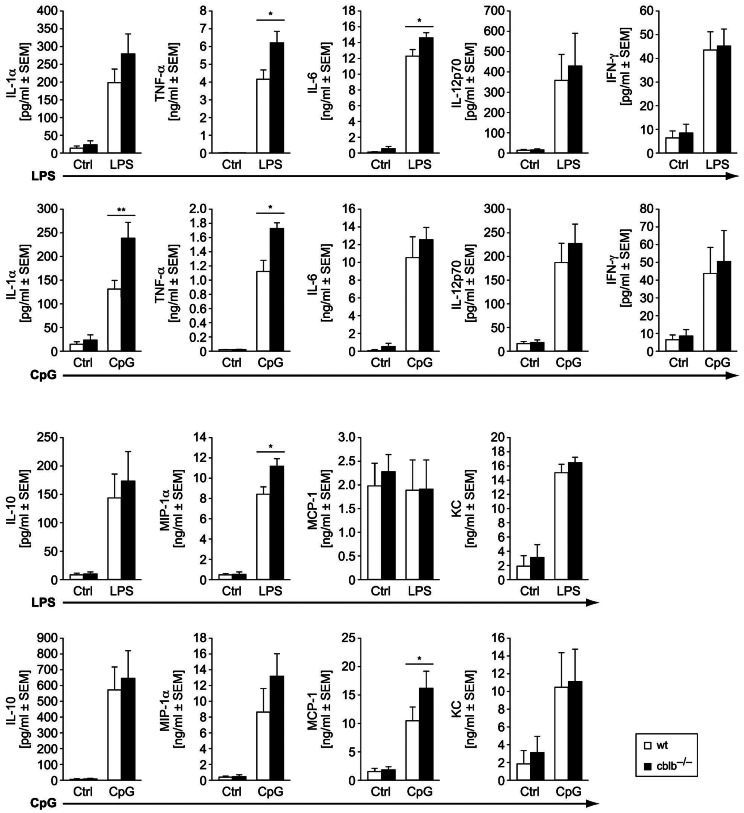
Cytokine and chemokine production by wildtype *versus cblb*−/− BMDs after stimulation with different TLR agonists. Wildtype and *cblb*−/− BMDCs were stimulated on day seven of culture with 1 µg/mL LPS or 100 nM CpG overnight for, IL-12p70, IL-10, IL-1α, IL-6, TNF-α, IFN-γ, KC, MIP-1α and MCP-1 measurement from cell culture supernatants were performed using Bioplex-Technology. Data represent mean value ± SEM of at least 4 independent experiments, *p<0.05 wildtype BMDCs *versus cblb*−/− BMDCs.

Production of cytokines and chemokines upon activation is a key feature of DC function [37], [38]. Therefore, we analyzed proinflammatory cytokine (IL-1α, IL-6, TNF-α, IL-10, IL-12p70, IFN-γ) and chemokine (KC, MCP-1, MIP-1α) secretion of *cblb*−/− BMDCs activated by different TLR agonists. Upon stimulation of wildtype and *cblb*−/− BMDCs by the TLR4 agonist LPS, significantly higher amounts of TNF-α, IL-6 and the chemokine MIP-1α could be detected in the supernatant of stimulated *cblb*−/− BMDCs (Figure 2). Only a slight and statistically not significant increase in IL-1α could be detected in LPS-stimulated *cblb*−/− BMDCs, whereas secretion of IFN-γ, IL-10, MCP-1 and CXCL1 (KC) was not affected by *cblb*-deficiency. Upon stimulation with the TLR-9 agonist CpG high amounts of the proinflammatory mediators IL-1α, TNF-α and the chemokine MCP-1 were detectable in *cblb*−/− *versus* wildtype BMDCs (Figure 2). In contrast, treating cells with the TLR3 agonist Poly(I:C) did not significantly alter their cytokine or chemokine secretion pattern (data not shown). Immature, non-stimulated BMDCs served as control and did not produce detectable amounts of cytokines (data not shown).

### Increased Allogeneic T cell Priming Potential of *cblb*−/− BMDCs

**Figure 3:**
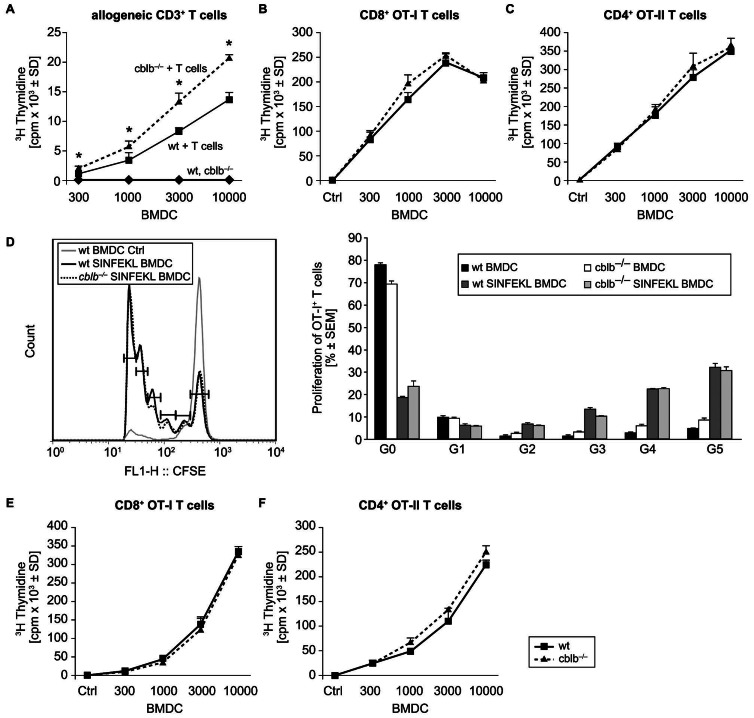
*Cblb*−/− BMDCs show increased allogeneic T cell proliferation but are not more effective in the induction of antigen-specific T cell responses and cross-presentation. (A) Increased allogeneic T cell proliferation induced by *cblb*−/− BMDCs. LPS matured wildtype or *cblb*−/− BMDCs were added in increasing numbers to 2×10^5^ allogeneic T cells for two days. Incorporation of ^3^H-Thymidine radioactivity during the last 16–18 hours of culture was measured. Data represent counts per minute (mean value ± SD, n = 4), representative of 4 independent experiments is shown, *p<0.05 wildtype BMDC *versus cblb*−/− BMDCs (Student t-test). (B/C) No difference in antigen specific T cell proliferation between wildtype and *cblb*−/− BMDCs. Wildtype and *cblb*−/− BMDCs were matured with 100 ng/ml LPS and primed with (B) 10 µM SIINFEKL-peptide (OT-I) or (C) 10 µM ISQAVHAAHAEINEAGR–peptide (OT-II) on day seven of culture overnight. Graded doses of BMDCs were then co-cultured with 2×10^5^ antigen-specific CD8^+^ (OT-I) or CD4^+^ (OT-II) transgenic T cells for 48 hours. Incorporation of ^3^H-Thymidine radioactivity during the last 16–18 hours of culture was measured. Data represent counts per minute (mean value ± SD, n = 3), representative of at least 3 independent experiments is shown. (D) Unaltered antigen specific T cell priming of *cblb*−/− BMDCs *in vivo*. C57BL/6 recipient mice were injected intravenously with 5×10^6^ CFSE-labeled transgenic CD8^+^ T cells. 24 hours thereafter 5×10^6^ wildtype *versus cblb*−/− BMDCs matured with 100 ng/ml LPS and pulsed *in vitro* with 10 µM SIINFKEL peptide or solvent as control were injected subcutanously in the flank of the mouse. Representative FACS histogram of CFSE dilution in OT-I transgenic T cells in the draining lymph node after injection of SIINFEKL peptide loaded wildtype *versus cblb*−/− BMDCs in wildtype recipients is shown. Data represent % proliferated OT-I^+^ T cells (mean value ± SEM, one representative experiment is shown with n = 3 mice per group), G = Generation. (E/F) No difference between wildtype and *cblb*−/− BMDCs in cross-presentation of protein antigen. Wildtype and *cblb*−/− BMDCs were cultured from day seven to day eight with 1 mg/ml OVA-protein and 100 ng/mL LPS. Graded doses of BMDCs were then co-cultured with 2×10^5^ antigen-specific (E) CD8^+^ (OT-I) or (F) CD4^+^ (OT-II) T cells for 48 hours. Incorporation of ^3^H-Thymidine radioactivity during the last 16 to 18 hours of culture was measured. Data are counts per minute (mean value ± SD, n = 2), representative of 2 independent experiments is shown.

Due to the increased production of cytokines, it is tempting to speculate that the T cell stimulatory capacity of *cblb*−/− BMDCs is modulated. Indeed,*cblb*−/− BMDCs were able to induce stronger allogeneic T cell priming capacity compared to wildtype BMDCs. To demonstrate this, T cells were prepared from spleens of BALB/c mice and co-cultured with LPS-matured wildtype or *cblb*−/− BMDCs. Increasing numbers of wildtype and *cblb*−/− BMDCs were incubated with a fixed number of allogeneic T cells for 48 hours. Our results in Figure 3A depict that BMDCs of *cblb*−/− mice are significantly more active MLR stimulators compared to wildtype BMDCs. Figure 3A shows that approximately 300 *cblb*−/− BMDCs could already trigger substantial T cell responses. The difference in induced T cell proliferation between wildtype and *cblb*−/− BMDCs increased with graded doses of BMDCs (Figure 3A). The proliferation responses by T cells alone or BMDCs alone served as control and were always low and not distinguishable between the genotypes.

### *In vitro* Antigen-specific T cell Proliferation not Altered by *cblb*−/− BMDCs

We next analyzed *in vitro* activation properties of *cblb*−/− BMDCs using T cell receptor transgenic mice, OVA-specific CD4^+^ (OT-II mice) and CD8^+^ (OT-I mice) T cells. Therefore SIINFEKL or ISQAVHAAHAEINEAGR peptide-pulsed BMDCs were cultured with OT-I or OT-II TCR transgenic T cells for two days. However, contrary to the MLR-data, *cblb*−/− BMDCs are not able to induce more effective antigen specific CD8^+^ or CD4^+^ T cell responses when compared to wildtype BMDCs (Figure 3B/C).

### *In vivo* Antigen-specific T cell Proliferation Unchanged by *cblb*−/− BMDCs

To verify the *in vitro* antigen-specific T cell proliferation results *in vivo*, we next analyzed the T cell-responses by intravenous injection of CFSE-labeled TCR transgenic OT-I/CD8^+^ T cells, by subsequent injection of peptide or non-pulsed wildtype or *cblb*−/− BMDCs. Three days thereafter, draining and non-draining lymph node, were evaluated for CD8^+^ T cell proliferation by CFSE dilution (Figure 3D). Most of the OT-I^+^ T cells in the draining lymph node entered cell cycle and underwent five cell divisions after injection of peptide-pulsed BMDCs, no matter if wildtype or *cblb*−/− BMDCs were applied. The unloaded BMDCs group elicited no proliferation and served as control group (Figure 3D). Equal to the *in vitro* results, no increased antigen-specific T cell proliferation could be induced by *cblb*−/− BMDCs *in vivo*, concluding that Cbl-b plays no relevant role in antigen-specific T cell priming in the OVA system tested here.

### Cross-presentation Capacity of *cblb*−/− BMDCs is not Affected

In the next set of experiments we used the protein antigen OVA to test the cross-presentation capacity of *cblb*−/− BMDCs. Therefore wildtype and *cblb*−/− BMDCs were incubated with soluble OVA-protein instead of peptides and *in vitro* cultured with OT-I CD8^+^and OT-II CD4^+^ TCR transgenic T cells for two days. *Cblb*−/− BMDCs induced proliferation rates comparable to wildtype BMDCs (Figure 3E/F). These results indicate that the induction of T cell responses by cross-presentation of OVA-peptides to the MHC-I receptor and the presentation of OVA-peptides to the MHC-II receptor is not affected by *cblb*-deficiency.

### Unaltered Macropinocytosis in *cblb*−/− BMDCs *versus* Wildtype BMDCs

**Figure 4:**
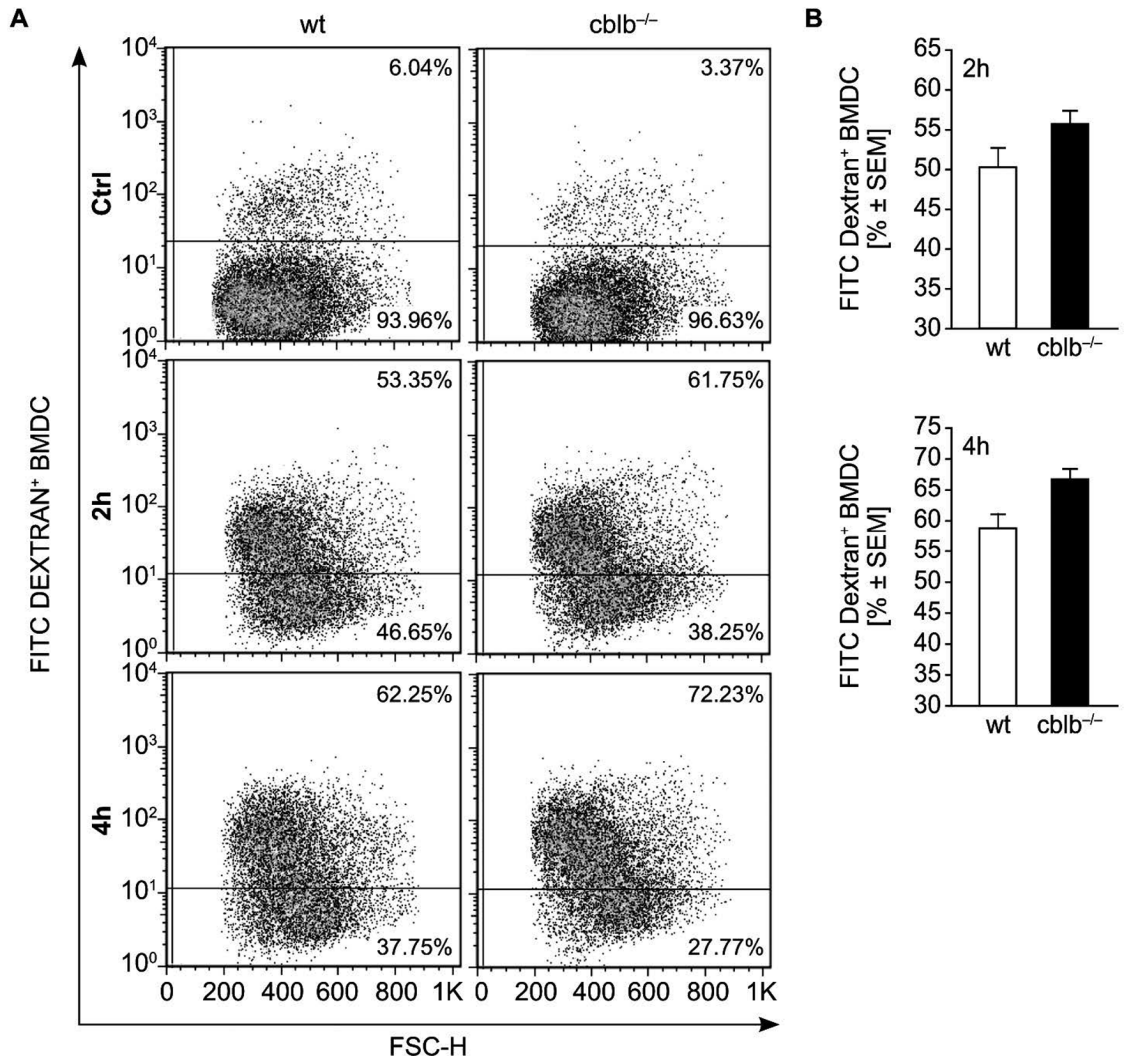
Macropinocytosis is not altered by *cblb*−/− deficiency. (A) Macropinocytosis was quantified by incubating immature day seven BMDCs with 2 mg/ml FITC-dextran at 37°C or at 4°C (negative control). After 2 and 4 hours, uptake was stopped and analyzed by FACS. Data represent mean value ± SEM; n = 4. (B) Representative dot plot of 4 independent experiments is shown.

We next analyzed the mechanisms of antigen uptake by BMDCs *via* macropinocytosis. Therefore immature BMDCs generated from either wildtype or *cblb*−/− animals were incubated with FITC Dextran and uptake was analyzed by flow cytometry (Figure 4A/B). Interestingly, immature BMDCs generated from *cblb*−/− mice showed comparable levels of FITC-dextran uptake when compared to wildtype BMDCs (Figure 4A/B). As we could detect upregulation of cytokine secretion by *cblb*−/− BMDCs (Figure 2), we additionally tested, whether these results could influence the ability of *cblb*−/− BMDCs to migrate from peripheral tissues to the T cell areas of draining lymphoid organs.

### Unaltered *cblb*−/− BMDCs Migration *in vivo*

**Figure 5:**
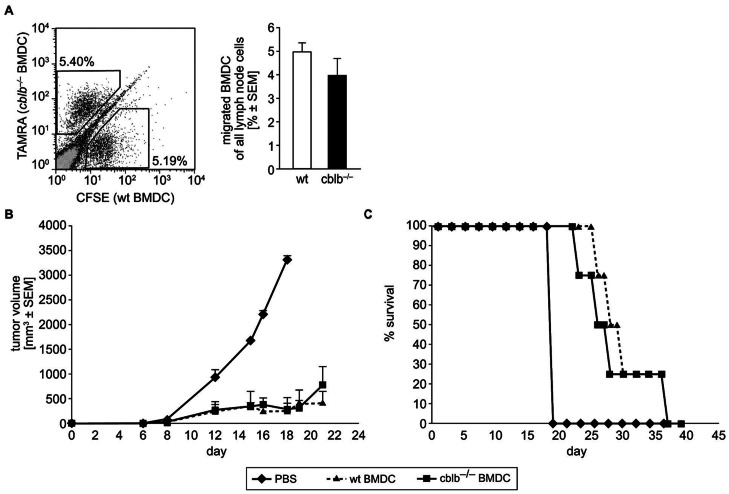
Migration capacity and therapeutic potential as tumor vaccine of *cblb*−/− BMDCs. (A) 0.125 mg of TB in FIA was injected in a total volume of 50 µl per hock into wildtype recipients. On day two CFSE labeled wildtype and TAMRA labeled *cblb*−/− BMDCs were injected in the hock in wildtype control recipients and TB injected recipients. 24 hours thereafter migration of the BMDCs in the draining lymph node and non-draining lymph node (not shown) was measured by flow cytometry. Data represent mean value ± SEM, n = 4 mice per group, two independent experiments. (B) 1×10^5^ B16-OVA cells were injected subcutaneously into the left flank of wildtype recipients. Tumor-bearing mice were subcutaneously vaccinated into the opposite right flank on day five with 2×10^5^ 10 µM SIINFEKL primed semi-mature wildtype *versus cblb*−/− BMDCs. Tumor growth was monitored thereafter every two/three days. Control animals received PBS. All data points represent tumor volume (mean value ± SEM, n = 4 mice), representative of two independent experiments is shown. (C) Survival of the same animals described in (B) was monitored.

Using an *in vivo* migration assay we investigated whether the absence of Cbl-b could influence the migration potential of BMDCs. Therefore, we induced peripheral inflammation by the injection of TB-FIA into the hock of mice and subsequently measured migration of differentially labeled wildtype and *cblb*−/− BMDCs to the draining lymph node. CFSE labeled wildtype and TAMRA labeled *cblb*−/− BMDCs were injected into the hock two days after induction of inflammation. 24 hours thereafter, migration of the dye-labeled BMDCs in the draining lymph node (Figure 5A) and non-draining lymph node as control was measured by flow cytometry. In all experiments, dye-labeled BMDCs were clearly detectable. BMDCs migrate best into the local draining (popliteal) lymph node and were not detectable in the inguinal lymph node. In solvent injected control mice the transferred BMDCs failed to migrate into the draining lymph node regardless of genotype. This migration assay revealed that both, *cblb*−/− and wildtype BMDCs were able to migrate properly to local draining lymph nodes (Figure 5A).

### *Cblb*−/− and Wildtype BMDCs have Equal Potential as Tumor Vaccine

To assess whether *cblb*-deficiency in BMDCs would increase their ability to infiltrate and reject tumors, fully immune-competent C57BL/6 wildtype mice were subcutaneously injected with B16-OVA melanoma cells and vaccinated with semi-matured SIINFEKL-loaded BMDCs from wildtype and *cblb*−/− donors on day five. Control groups received PBS solvent only. While at day 24 all mice in the untreated group had to be sacrificed due to large tumor sizes, treatment with SIINFEKL-loaded wildtype or *cblb*−/− BMDCs substantially delayed tumor outgrowth (Figure 5B). However, tumor growth suppression and survival was comparable in mice receiving *cblb*−/− BMDCs compared to wildtype BMDCs (Figure 5B/C).

## Discussion

The here presented study is based on a recently published article of Shin-Heng Chiou et al., who postulated that the E3 ligase c-Cbl plays a critical role in DC biology [9]. They identified a proinflammatory phenotype of *ccbl*−/− *versus* wildtype BMDCs, which is accompanied with an increased potency as DC-based vaccine against established tumors. So far, it was entirely unknown whether Cbl-b is expressed and – if so – modulates DC biology. Therefore, we have systematically compared wildtype with *cblb*−/− BMDCs and conclude from our data that Cbl-b has only limited impact on the functional properties of murine BMDCs.

Our results show that Cbl-b protein is highly expressed in murine BMDCs. Absence of Cbl-b does not inhibit BMDC differentiation *in vitro*, as we were not able to detect altered differentiation of BMDCs in a seven day culture when compared to wildtype BMDCs. When we focused on the phenotype of *cblb*−/− BMDCs however, we were able to detect a significant and consistent increase of the endocytotic receptor DEC-205 in *cblb*−/− BMDCs, while other DC markers, such as CD206, CD83 and CD11b or the DC activation markers MHC class II, CD40, CD80 and CD86 were not differentially expressed.

A hallmark of DC function is the paracrine regulation of immune cells by production of cytokines and chemokines such as IL-1α, IL-1β, TNF-α, IL-6 MIP-1α, and β [39]–[41]. One mechanism is pathogen recognition by TLRs, which provokes rapid activation of the immune system by inducing expression of proinflammatory cytokines. It has recently been demonstrated that E3 ubiquitin ligase Cbl-b degrades MyD88, a critical component of TLR-signaling [10]–[13]. Whereas the stimulation of TLR4 and TLR9 mainly facilitates the activation of the MyD88 dependent pathway which leads to the production of inflammatory cytokines, TLR3 signaling seems to transducer its signals mainly through the MyD88-independent pathway [42], [43]. According to this concept, we here show that there is a general trend towards higher cytokine production upon stimulation of DCs with LPS or CpG, reaching statistical significance for IL-1α, TNF-α for both TLR-agonists as well as IL-6 and MIP-1α for LPS and MCP-1 for CpG. This observation is in line with the paper of Shin-Heng Chiou et al., which showed increased proinflammatory cytokine secretion of *ccbl*−/− BMDCs after TLR stimulation. However, no alterations in TLR3 mediated cytokine expression could be observed between wt and *cblb*−/− BMDCs, which further confirms that Cbl-b degrades MyD88 and the subsequent suppression of MyD88 dependent inflammatory responses. Thus Cbl-b might function as a negative regulator of TLR triggered inflammatory responses by directly inhibiting and degrading MyD88 [10].

Interestingly, *cblb*−/− BMDCs are more effective inducers of an allogeneic T cell proliferation in MLR cultures, whereas their potential to induce antigen-specific T cell responses against both, MHC class I- or class II-restricted OVA-peptides, was not affected. Whereas the increased levels of proinflammatory cytokines and chemokines could be linked to an increased allogeneic T cell proliferation, the peptide-induced T cell proliferation seems to be less sensitive of the modulated cytokine milieu. This could be due to the fact that OT-I and OT-II T cells may already be committed to activation or proliferation by just minor stimuli, where very low concentrations of Ag are sufficient to induce strong proliferation. Therefore the increase in cytokine production by *cblb*−/− BMDCs may not be sufficient to influence OT-I and OT-II T cells in their peptide-induced proliferation response. Activation of DCs also stimulates T cells to produce a variety of cytokines, and recent studies suggest that some of these cytokines may provide, next to co-stimulatory ligands and the Ag presented by DCs, an essential third signal that is required to fully activate T cells and avoid tolerance induction.Recent reports further showed that stimulation of CD8^+^ T cells with a high level of Ag in the presence of co-stimulation can lead to strong *in vitro* proliferation but the cells fail to develop effector function unless signal 3 in the form of the cytokine IL-12 is also provided. Thus, *cblb*−/− BMDCs are not different from wt BMDCs with regard to the induction of Ag-specific clonal expansion but may modify other functions, such as induction of tolerance or effector functions [44], [45], which we have not specifically addressed in this report.

To explore whether these *in vitro* observations also hold true *in vivo*, we next tested the T cell proliferation capacity of adoptively transferred TCR transgenic CD8^+^/OT-I T cells, in response to SIINFEKL peptide loaded wildtype *versus cblb*−/− BMDCs. Consistent with the results from our *in vitro* studies, we were not able to detect a difference of the *in vivo* T cell proliferation induced by wt or *cblb*−/− BMDCs.

As cross-presentation provides the immune system with an important mechanism for generating immunity to viruses and tolerance to self, we further tested if *cblb*−/− BMDCs could incorporate and process proteins *via* the endocytotic machinery [46]. Using this approach, we were able to demonstrate that the processing of proteins to MHC class I and class II seems not to be impaired in *cblb*−/− BMDCs. This result correlates with our surface marker characterisation of *cblb*−/− BMDCs, as no alteration in the expression of the CD206 receptor could be observed and it has been described that the mannose receptor is an uptake receptor for OVA protein [47].

Capture of Antigens by surface receptors such as Fc-Receptors, Mannose Receptors (e.g. CD206) or DEC-205 receptors allows efficient delivery of the antigen to the processing compartment *via* receptor-mediated endocytosis [48]–[50]. Antigens that fail to bind to cell surface receptors can still be taken up by fluid phase pinocytosis and presented by antigen presenting cells (APC) but with much lower efficiency [51]. Of note, macropinocytosis was not statistically different between *cblb*−/− and wt BMDCs. However, it is also conceivable that DEC-205 expression in DCs might be relevant for steady state function of DCs, as DEC-205 for example has been shown to be associated with regulatory T cell induction thereby regulating peripheral tolerance [52]–[55].

The capacity of DCs to migrate into T cell areas of lymph nodes is a key event in initiating immunity, and it may represent a critical step for the induction of an anti-tumor immune response. DCs are also known to produce different immune stimulatory cytokines and chemokines thereby regulating the traffic of Th1 and Th2 cells into inflamed tissue. Thus we further questioned if Cbl-b could play a role in the migration potential of DCs into inflamed tissue [38]. Here we could clearly show that *cblb*−/− BMDCs are as potent in migration to local lymph nodes after subcutaneous injection as wildtype BMDCs are. The data are in agreement with the observation that the critical lymph node-homing receptor for DC-migration from the periphery into the lymph node CCR7 was not affected by *cblb*-deficiency (data not shown) [56], [57].

We next explored whether Cbl-b deficiency might improve therapeutic potential of a BMDC vaccine applied in the B16-OVA model. However the vaccination of tumor-bearing animals with SIINFEKL-pulsed *cblb*−/− BMDCs showed no difference, neither in tumor regression nor in survival rates when compared to wildtype BMDC application. In contrast to the data from c-Cbl deleted mice we conclude from these experiments that despite higher DEC-205 expression and an increased expression of proinflammatory cytokines and chemokines upon TLR-stimulation, the concept of Cbl-b targeting alone in DCs is not suitable to increase the efficacy of DC vaccines *in vivo*.

## References

[1] Steinman RM (2012) Decisions about dendritic cells: past, present, and future. Annual Review of Immunology 30: 1–22. 10.1146/annurev-immunol-100311-102839 22136168

[2] Den Brok MHMGM, Nierkens S, Figdor CG, Ruers TJM, Adema GJ (2005) Dendritic cells: tools and targets for antitumor vaccination. Expert Review of Vaccines 4: 699–710. 16221071 10.1586/14760584.4.5.699

[3] Tjoa BA, Simmons SJ, Bowes VA, Ragde H, Rogers M, et al (1998) Evaluation of phase I/II clinical trials in prostate cancer with dendritic cells and PSMA peptides. The Prostate 36: 39–44. 9650914 10.1002/(sici)1097-0045(19980615)36:1<39::aid-pros6>3.0.co;2-6

[4] Engell-Noerregaard L, Hansen TH, Andersen MH, Thor Straten P, Svane IM (2009) Review of clinical studies on dendritic cell-based vaccination of patients with malignant melanoma: assessment of correlation between clinical response and vaccine parameters. Cancer Immunology, Immunotherapy 58: 1–14. 18719915 10.1007/s00262-008-0568-4 PMC11030652

[5] Delamarre L, Mellman I (2011) Harnessing dendritic cells for immunotherapy. Seminars in Immunology 23: 2–11. 21377379 10.1016/j.smim.2011.02.001

[6] Schuler G (2010) Dendritic cells in cancer immunotherapy. European Journal of Immunology 40: 2123–2130. 20853498 10.1002/eji.201040630

[7] Nau MM, Lipkowitz S (2003) Comparative genomic organization of the cbl genes. Gene 308: 103–113. 12711395 10.1016/s0378-1119(03)00471-2

[8] Wallner S, Gruber T, Baier G, Wolf D (2012) Targeting Cbl-b to enhance lymphocyte effector function. Developmental and Clinical Immunology 2012: 1–5. 10.1155/2012/692639 PMC3328896 22550535

[9] Chiou S-H, Shahi P, Wagner RT, Hu H, Lapteva N, et al.. (2011) The E3 ligase c-Cbl regulates dendritic cell activation. EMBO reports: 1–9. 10.1038/embor.2011.143 PMC3166462 21799517

[10] Han C, Jin J, Xu S, Liu H, Li N, et al (2010) Integrin CD11b negatively regulates TLR-triggered inflammatory responses by activating Syk and promoting degradation of MyD88 and TRIF via Cbl-b. Nature Immunology 11: 734–742. 20639876 10.1038/ni.1908

[11] Kaisho T, Akira S (2002) Toll-like receptors as adjuvant receptors. Biochimica et Biophysica Acta 1589: 1–13. 11909637 10.1016/s0167-4889(01)00182-3

[12] Takeda K, Kaisho T, Akira S (2003) Toll-like receptors. Annual Review of Immunology 21: 335–376. 10.1146/annurev.immunol.21.120601.141126 12524386

[13] Takeda K (2005) Toll-like Receptors and their Adaptors in Innate Immunity. Science: 3–11.

[14] Nurieva R, Thomas S, Nguyen T, Martin-Orozco N, Wang Y, et al (2006) T-cell tolerance or function is determined by combinatorial costimulatory signals. The EMBO Journal 25: 2623–2633. 16724117 10.1038/sj.emboj.7601146 PMC1478197

[15] Karwacz K, Bricogne C, MacDonald D, Arce F, Bennett CL, et al (2011) PD-L1 co-stimulation contributes to ligand-induced T cell receptor down-modulation on CD8^+^ T cells. EMBO Molecular Medicine 3: 581–592. 21739608 10.1002/emmm.201100165 PMC3191120

[16] Brahmer JR, Tykodi SS, Chow LQM, Hwu W-J, Topalian SL, et al (2013) Safety and Activity of Anti-PD-L1 Antibody in Patients with Advanced Cancer. New England Journal of Medicine 366: 2455–2465. 10.1056/NEJMoa1200694 PMC3563263 22658128

[17] Topalian SL, Hodi FS, Brahmer JR, Gettinger SN, Smith DC, et al (2012) Safety, Activity, and Immune Correlates of Anti-PD-1 Antibody in Cancer. New England Journal of Medicine 366: 2443–2454. 22658127 10.1056/NEJMoa1200690 PMC3544539

[18] Heissmeyer V, Macián F, Im S-H, Varma R, Feske S, et al (2004) Calcineurin imposes T cell unresponsiveness through targeted proteolysis of signaling proteins. Nature Immunology 5: 255–265. 14973438 10.1038/ni1047

[19] Jeon M-S, Atfield A, Venuprasad K, Krawczyk C, Sarao R, et al (2004) Essential role of the E3 ubiquitin ligase Cbl-b in T cell anergy induction. Immunity 21: 167–177. 15308098 10.1016/j.immuni.2004.07.013

[20] Kojo S, Elly C, Harada Y, Langdon WY, Kronenberg M, et al (2009) Mechanisms of NKT cell anergy induction involve Cbl-b-promoted monoubiquitination of CARMA1. Proceedings of the National Academy of Sciences of the United States of America 106: 17847–17851. 19815501 10.1073/pnas.0904078106 PMC2764888

[21] Chiang YJ, Kole HK, Brown K, Naramura M, Fukuhara S, et al (2000) Cbl-b regulates the CD28 dependence of T-cell activation. Nature 403: 216–220. 10646609 10.1038/35003235

[22] Paolino M, Thien CBF, Gruber T, Hinterleitner R, Baier G, et al (2011) Essential role of E3 ubiquitin ligase activity in Cbl-b-regulated T cell functions. Journal of Immunology 186: 2138–2147. 10.4049/jimmunol.1003390 21248250

[23] Loeser S, Loser K, Bijker MS, Rangachari M, van der Burg SH, et al (2007) Spontaneous tumor rejection by cbl-b-deficient CD8^+^ T cells. The Journal of Experimental Medicine 204: 879–891. 17403934 10.1084/jem.20061699 PMC2118550

[24] Bachmaier K, Krawczyk C, Kozieradzki I, Kong Y-Y, Sasaki T, et al (2000) Negative regulation of lymphocyte activation and autoimmunity by the molecular adaptor Cbl-b. Nature 403: 211–216. 10646608 10.1038/35003228

[25] Krawczyk CM, Jones RG, Atfield A, Bachmaier K, Arya S, et al (2005) Differential control of CD28-regulated in vivo immunity by the E3 ligase Cbl-b. The Journal of Immunology 174: 1472–1478. 15661906 10.4049/jimmunol.174.3.1472

[26] Wohlfert EA, Callahan MK, Clark RB (2004) Resistance to CD4^+^CD25^+^ regulatory T cells and TGF-β in Cbl-b−/− mice. Journal of Immunology 173: 1059–1065. 10.4049/jimmunol.173.2.1059 15240694

[27] Wohlfert EA, Gorelik L, Mittler R, Flavell RA, Clark RB (2006) Cutting Edge: Deficiency in the E3 Ubiquitin Ligase Cbl-b Results in a Multifunctional Defect in T Cell TGF-β Sensitivity in Vitro and In Vivo. Journal of Immunology 176: 1316–1320. 10.4049/jimmunol.176.3.1316 16424156

[28] Lutz-Nicoladoni C, Wallner S, Stoitzner P, Pircher M, Gruber T, et al (2011) Reinforcement of cancer immunotherapy by adoptive transfer of cblb-deficient CD8^+^ T cells combined with a DC vaccine. Immunology and Cell Biology 2011: 1–5. 10.1038/icb.2011.11 21383769

[29] Purev E, Neff L, Horne WC, Baron R (2009) c-Cbl and Cbl-b Act Redundantly to Protect Osteoclasts from Apoptosis and to Displace HDAC6 from β-Tubulin, Stabilizing Microtubules and Podosomes. Molecular Biology of the Cell 20: 4021–4030. 19641021 10.1091/mbc.E09-03-0248 PMC2743621

[30] Huang F, Gu H (2008) Negative regulation of lymphocyte development and function by the Cbl family of proteins. Immunological Reviews 224: 229–238. 18759930 10.1111/j.1600-065X.2008.00655.x

[31] Lutz MB, Kukutsch N, Ogilvie ALJ, Roßner S, Koch F, et al (1999) An advanced culture method for generating large quantities of highly pure dendritic cells from mouse bone marrow. Journal of Immunological Methods 223: 77–92. 10037236 10.1016/s0022-1759(98)00204-x

[32] McLellan AD (2002) Anatomic location and T-cell stimulatory functions of mouse dendritic cell subsets defined by CD4 and CD8 expression. Blood 99: 2084–2093. 11877283 10.1182/blood.v99.6.2084

[33] Lyons AB (2000) Analysing cell division in vivo and in vitro using flow cytometric measurement of CFSE dye dilution. Journal of Immunological Methods 243: 147–154. 10986412 10.1016/s0022-1759(00)00231-3

[34] Kamala T (2007) Hock immunization: A humane alternative to mouse footpad injections. Journal Immunology Methods 328: 204–214. 10.1016/j.jim.2007.08.004 PMC2464360 17804011

[35] Lugade AA, Moran JP, Gerber SA, Rose RC, Frelinger JG, et al.. (2012) Local radiation therapy of B16 melanoma tumors increases the generation of tumor antigen-specific effector cells that traffic to the tumor. The Journal of Immunology: 7516–7523. 10.4049/jimmunol.174.12.7516 15944250

[36] Bauer B, Krumböck N, Ghaffari-Tabrizi N, Kampfer S, Villunger A, et al (2000) T cell expressed PKCtheta demonstrates cell-type selective function. European Journal of Immunology 30: 3645–3654. 11169407 10.1002/1521-4141(200012)30:12<3645::AID-IMMU3645>3.0.CO;2-#

[37] Morelli AE (2001) Cytokine production by mouse myeloid dendritic cells in relation to differentiation and terminal maturation induced by lipopolysaccharide or CD40 ligation. Blood 98: 1512–1523. 11520802 10.1182/blood.v98.5.1512

[38] Moser B, Loetscher P (2001) Lymphocyte traffic control by chemokines. Nature Immunology 2: 123–128. 11175804 10.1038/84219

[39] Roake JA, Rao AS, Morris PJ, Larsen CP, Hankins DF, et al (1995) Dendritic Cell Loss from Nonlymphoid Tissues after Systemic Administration of Lipopolysaccharide, Tumor Necrosis Factor, and Interleukin 1. Journal of Experimental Medicien 181: 2237–2247. 10.1084/jem.181.6.2237 PMC2192059 7760009

[40] Idzko M, Panther E, Stratz C, Müller T, Bayer H, et al (2004) The serotoninergic receptors of human dendritic cells: identification and coupling to cytokine release. Journal of Immunology 172: 6011–6019. 10.4049/jimmunol.172.10.6011 15128784

[41] O’Connell PJ, Wang X, Leon-Ponte M, Griffiths C, Pingle SC, et al (2006) A novel form of immune signaling revealed by transmission of the inflammatory mediator serotonin between dendritic cells and T cells. Blood 107: 1010–1017. 16223770 10.1182/blood-2005-07-2903 PMC1895901

[42] Medzhitov R (2001) Toll-like receptors and innate immunity. Nature Reviews Immunology 1: 135–145. 10.1038/35100529 11905821

[43] Akira S, Takeda K (2004) Toll-like receptor signalling. Nature Reviews Immunology 4: 499–511. 10.1038/nri1391 15229469

[44] Curtsinger JM, Lins DC, Mescher MF (2003) Signal 3 Determines Tolerance versus full Activation of naive CD8 T cells: Dissociating Proliferation and Development of Effector Function. The Journal of Experimental Medicine 197: 1141–1151. 12732656 10.1084/jem.20021910 PMC2193970

[45] Liechtenstein T, Dufait I, Lanna A, Breckpot K, Escors D (2013) Modulating co-stimulation during antigen presentation to enhance cancer immunotherapy. Immunology‚ Endocrine & Metabolic Agents in Medicinal Chemistry 12: 1–23. 10.2174/187152212802001875 PMC3428911 22945252

[46] Heath WR, Carbone FR (2001) Cross-presentation in viral immunity and self-tolerance. Nature Reviews Immunology 1: 126–134. 10.1038/35100512 11905820

[47] Burgdorf S, Lukacs-kornek V, Kurts C (2012) The Mannose Receptor Mediates Uptake of Soluble but Not of Cell-Associated Antigen for Cross-Presentation. The Journal of Immunology 176: 6770–6776. 10.4049/jimmunol.176.11.6770 16709836

[48] Sallusto F, Lanzavecchia A (1994) Efficient Presentation of Soluble Antigen by Cultured Human Dendritic Cells Is Maintained by Granulocyte/Macrophage Colony-stimulating Factor Plus Interleukin 4 and Downregulated by Tumor Necrosis Factor alpha. Journal of Experimental Medicine 179: 1109–1118. 8145033 10.1084/jem.179.4.1109 PMC2191432

[49] Sallusto F, Cella M, Danieli C, Lanzavecchia A (1995) Dendritic Cells Use Macropinocytosis and the Mannose Receptor to Concentrate Macromolecules in the Major Histocompatibility Complex Class II Compartment: Downregulation by Cytokines and Bacterial Products. Journal of Experimental Medicine 182: 389–400. 7629501 10.1084/jem.182.2.389 PMC2192110

[50] Wanping J, William JS, Christine H, Michael P, Asra M, et al (1995) The receptor DEC205 expressed by DC and thymic epithelial cells is involved in antigen processing. Nature 375: 151–155. 7753172 10.1038/375151a0

[51] Lanzavecchia A (1990) Receptor-Mediated Antigen Uptake and its Effect on Antigen Presentation to Class II-Restricted T Lymphocytes. Annual Review of Immunology 8: 773–793. 10.1146/annurev.iy.08.040190.004013 2188679

[52] Mukhopadhaya A, Hanafusa T, Jarchum I, Chen Y-G, Iwai Y, et al (2008) Selective delivery of β cell antigen to dendritic cells in vivo leads to deletion and tolerance of autoreactive CD8^+^ T cells in NOD mice. Proceedings of the National Academy of Sciences of the United States of America 105: 6374–6379. 18430797 10.1073/pnas.0802644105 PMC2359791

[53] Mahnke K, Qian Y, Knop J, Enk AH (2003) Induction of CD4^+^/CD25^+^ regulatory T cells by targeting of antigens to immature dendritic cells. Blood 101: 4862–4869. 12543858 10.1182/blood-2002-10-3229

[54] Bonifaz L, Bonnyay D, Mahnke K, Rivera M, Nussenzweig MC, et al (2002) Efficient Targeting of Protein Antigen to the Dendritic Cell Receptor DEC-205 in the Steady State Leads to Antigen Presentation on Major Histocompatibility Complex Class I Products and Peripheral CD8^+^ T Cell Tolerance. Journal of Experimental Medicine 196: 1627–1638. 12486105 10.1084/jem.20021598 PMC2196060

[55] Hawiger D, Inaba K, Dorsett Y, Guo M, Mahnke K, et al (2001) Dendritic cells induce peripheral T cell unresponsiveness under steady state conditions in vivo. The Journal of Experimental Medicine 194: 769–779. 11560993 10.1084/jem.194.6.769 PMC2195961

[56] Ohl L, Mohaupt M, Czeloth N, Hintzen G, Kiafard Z, et al (2004) CCR7 Governs Skin Dendritic Cell Migration under Inflammatory and Steady-State Conditions. Cell 21: 279–288. 10.1016/j.immuni.2004.06.014 15308107

[57] Förster R, Schubel A, Breitfeld D, Kremmer E, Renner-Müller I, et al (1999) CCR7 coordinates the primary immune response by establishing functional microenvironments in secondary lymphoid organs. Cell 99: 23–33. 10520991 10.1016/s0092-8674(00)80059-8

